# The accuracy of multiparametric MRI in men with negative biopsy and elevated PSA level—Can it rule out clinically significant prostate cancer?

**DOI:** 10.1016/j.urolonc.2013.06.007

**Published:** 2014-01

**Authors:** Mohamed Abd-Alazeez, Hashim U. Ahmed, Manit Arya, Susan C. Charman, Eleni Anastasiadis, Alex Freeman, Mark Emberton, Alex Kirkham

**Affiliations:** a Department of Urology, University College London Hospitals NHS Foundation Trust, London, UK; b Department of Urology, Faculty of medicine, Fayoum University, Fayoum, Egypt; c Division of Surgery and Interventional Science, University College London, London, UK; d Barts Cancer Institute, Queen Mary, University of London, London, UK; e Department of Health Services Research and Policy, London School of Hygiene and Tropical Medicine; f Clinical Effectiveness Unit, Royal College of Surgeons of England, London, UK; g Department of Histopathology, University College Hospitals NHS Foundation Trust, London, UK; h Department of Radiology, University College Hospitals NHS Foundation Trust, London, UK

**Keywords:** Clinically significant disease, Multiparametric MRI, negative biopsy, Template prostate mapping

## Abstract

**Purpose:**

To assess the performance of multiparametric magnetic resonance imaging (mp-MRI) in patients with previous negative transrectal ultrasound (TRUS) guided prostate biopsy.

**Materials and methods:**

Fifty-four patients with at least 1 previous negative TRUS prostate biopsy underwent mp-MRI in the form of T2-weighted, diffusion-weighted, and dynamic contrast-enhanced imaging. This was followed by transperineal template systematic prostate biopsies. Analysis was done based on 2 sectors per prostate, right and left (108 sectors out of 54 prostates). mp-MRI was scored using an ordinal scale 1 to 5 based on the suspicion of the presence of clinically significant disease. We used 6 different definitions for clinically significant disease and tested the performance of mp-MRI at each single definition.

**Results:**

Median age was 64 (range, 39–75), median PSA level was 10 (range, 2–23), and median number of biopsies was 45 (range, 21–137). Cancer of any volume and any grade was detected in 34 of 54 (63%) patients. mp-MRI accuracy at detection of clinically significant cancer using University College London (UCL) definition 2 (any Gleason score of 4 or maximum cancer core length of ≥4 mm or both) showed sensitivity of 76%, specificity of 42%, positive predictive value of 38%, and negative predictive value of 79%. For a different definition of significant tumor (UCL definition 1; dominant Gleason score 4 or maximum cancer core length ≥6 mm or both), the sensitivity was 90%, specificity 42%, positive predictive value 26%, and negative predictive value 95%.

**Conclusions:**

mp-MRI showed good performance at both detection and ruling out clinically significant disease, according to the definition used. mp-MRI can then be used as a triage test in the population with persistently elevated or rising PSA levels to select patients that can avoid unnecessary prostate biopsy.

## 1 Introduction

We know that transrectal biopsy misses significant prostate cancers, because of both a random sampling error (repeat biopsies using the same technique will detect tumor in around a quarter [1]), and because between a quarter and a third of significant tumors lie in the anterior part of the gland, based on studies of radical prostatectomy specimens [2].

Several studies have recently documented an incidence of tumors in men with a negative biopsy but persistently elevated PSA level. A study using systematic transperineal mapping biopsy showed tumor in 57%, with the majority of positive cores lying anteriorly [3]. Others have found tumor in 40% [4] and 59% [5] of men when magnetic resonance imaging (MRI) findings are used to target biopsy.

Recent data have suggested that most significant (defined as>0.5 mL) tumors are detected on MRI, including those in the anterior part of the gland [6,7], with diffusion especially effective in the latter [4]. Our aim was to assess the performance of multiparametric MRI (mp-MRI) in men with a continuing suspicion of tumor but negative TRUS biopsy, by prospective comparison of MRI findings with *systematic* transperineal mapping biopsies. Such mapping biopsies have a high sensitivity for significant disease and are the best method we have for confirming absence of disease within the prostate: men without tumor would rarely be subjected to prostatectomy [8].

## 2 Material and methods

Research ethics committee exemption was granted for this single institution study. A total of 58 men with at least 1 negative TRUS-guided prostate biopsy underwent mp-MRI (index test) followed by template prostate mapping biopsy (reference standard). Four men were excluded from the study as they received limited template biopsy (less than 20 cores were taken). This gives a total number of 54 patients included in the study. Patients had between 1 and 3 prior negative biopsies (33 had previous 1 negative set of biopsies, 16 had previous 2 negative sets of biopsies, and 5 had previous 3 negative sets of biopsies). Most of the patients included in the study were referred from other health care centers to our tertiary referral hospital. Although we do not have a complete record of the number of cores taken during each biopsy at the peripheral centers, it is considered standard practice at the referring units to take at least 10 to 12 core biopsies.

All patients included in the study had either increasing or persistently high PSA level.

### 2.1 MRI (index test)

MRI comprised T2-weighted (T2W), diffusion-weighted (DW), and dynamic contrast-enhanced (DCE) imaging with either 1.5 T (Siemens Avanto, *n* = 49) or 3 T (Philips Achieva, *n* = 5) machines. In each case, a multichannel pelvic-phased array coil was used. Contrast was gadoterate meglumine (*dotarem*, Guerbet, France) at a dose of at least 0.1 mmol/kg, administered at 3 mL/s. The sequence parameters for the 1.5-T scans are shown in Table 1; each specification was exceeded at 3 T, and in each case the scans conformed to the recommended parameters of the recent European Society of Urogenital Radiology guidelines on mp-MRI [9].

Eight radiologists, of 3 to 8 years of experience, reported all the mp-MR images using a score from 1 to 5 as in the recent European Guidelines [9]. In 5 cases, the initial report did not contain a numerical score, and 1 was generated by one radiologist (A.K.) based on the report text only. mp-MRI was performed in a blinded manner to the template biopsy as all imaging reports were committed to the electronic medical record before the biopsy result became available.

### 2.2 Template biopsy (reference standard)

All patients underwent systematic template prostate mapping biopsy using a brachytherapy grid under general anesthesia in the method previously described by Barzell and Melamed [10]. Basal and apical cores were obtained routinely, and the minimum number of samples was 20.

For assessment of the performance of MRI, we analyzed the prostate in halves (right and left), so that there were 108 sectors (54 patients). To determine the proportion of tumors lying anteriorly on histology, we considered anterior cores to lie in front of an imaginary transverse line drawn through the urethra at midgland level. Biopsy cores taken from the lateral part of the prostate (according to Barzell's definitions) were considered posterior. Whenever a suspected lesion crossed the midline on MRI, both prostate halves were attributed the same scoring for that lesion.

Fifteen patients had additional targeted biopsies besides the systematic template prostate biopsies. This was done based on cognitive registration. In other words, the operator took biopsies from the 20 Barzell zones then took additional biopsies from an area of the prostate deemed to be suspicious or harbor cancer on MRI (score ≥ 3).

### 2.3 Target conditions (Table 2)

The pathological outputs from the reference test were grouped into a number of definitions of clinical significance, or target conditions [11,12] to reflect the fact that no universally accepted definition currently exists. The histological reporting in our institution follows the classic scheme of interpreting the Gleason grading, the one used before the International Society of Urological Pathology 2005 guidelines [13]. In other words, Gleason scoring was based on the most frequent pattern and not the highest grade detected on histological analysis. Further, the cancer core length was reported as the actual amount of cancer seen in each core without counting the intervening areas of benign glands [14].

### 2.4 Statistical analysis

An mp-MRI score of ≥3 was used to designate a positive index test for the purpose of ruling out clinically significant disease. The effect of varying this threshold to ≥4 was also evaluated for predicting clinically significant disease. If the mp-MRI was positive in a sector proven to harbor clinically insignificant disease, according to the definition used, this area was deemed as a false positive. Accuracy figures (sensitivity, specificity, and positive and negative predictive values), together with 95% confidence intervals (95% CIs), were calculated for the performance of mp-MRI at each definition of significant disease.

### 2.5 Primary objective

To assess the ability of mp-MRI (with a score of ≥3/5 considered positive) to detect UCL definition 2 of clinically significant disease, defined as any cancer with Gleason pattern 4 or greater (≥3 + 4) or maximum cancer core length ≥4 mm or both.

### 2.6 Secondary objectives

First, to examine the performance of the test when: (1) varying the histological definition of clinically significant disease and (2) varying the MRI threshold of positivity from 3/5 to 4/5. Second, we aimed to determine the distribution of tumors found at template biopsy—in particular, the proportion that lay anterior to the urethra.

## 3 Results

Baseline demographic data are shown in Table 3.

Cancer of any grade or burden was identified in 34 of 54 (63%) patients.

mp-MRI was ≥3 in 45 patients. Targeted biopsies (range 2–9) were taken in 15 patients and showed cancer in 8 of 16 (53%) patients. None of those patients had cancer only on the targeted area.

Of 34 patients with a positive biopsy, 16 patients (47%) had an anterior tumor only, 3 (9%) had a posterior tumor only, and 15 (44%) had a tumor in both anterior and posterior cores.

Table 4 illustrates the correlation between different MRI scores and systematic template mapping biopsy findings based on Gleason grade.

### 3.1 Primary outcome

In ruling out the presence of any Gleason score ≥4 or maximum cancer core length ≥4 mm (UCL definition 2) or both, mp-MRI had a sensitivity and negative predictive value (NPV) of 76% (95% CI, 59%–91%) and 79% (95% CI, 66%–92%), respectively (Table 5).

### 3.2 Secondary outcomes

The performance of the test for different levels of clinical significance and at different MRI thresholds is shown in Tables 6 and 7. At mp-MRI threshold of ≥3, both sensitivity and NPV for detection of cancers of Gleason score ≥4 + 3 were 100% (95% CI, 100%–100%), whereas those for Gleason score ≥3 + 4 were 87% (95% CI, 70%–100%) and 92% (95% CI, 82%–100%), respectively.

## 4 Discussion

The use of systematic transperineal biopsy for verification enabled us to estimate the sensitivity and negative predictive value of mp-MRI in a group of men with previous negative biopsy—something that has been difficult in most previous studies that have used targeted biopsy. Our main finding was of a sensitivity of 76% for clinically significant disease (UCL Def 2), based on an analysis at half-gland level, with sensitivity of 87% for any Gleason 4 element and 100% for tumors with dominant Gleason 4. Specificity was fairly low (38%–46%), though improved if a threshold of 4/5 was used to define positive disease (71%–86%). However, using a score of 4/5 (“likely” rather than “equivocal”) as the definition for significant disease means that even some tumors with dominant Gleason 4 elements would be missed.

It is becoming clear that small amounts of low-grade tumor are both unlikely to harm the patient [12] and unlikely to be detected on MRI [7]. Because the definitions of clinically significant disease vary so much, we analyzed the performance of MRI at a number of levels. The appropriate definition of course varies with the patient: in a relatively old man with comorbidities, exclusion of dominant Gleason 4 disease may be all that is necessary, whereas in a younger man, the exclusion of UCL definition 2 (<4 mm and no Gleason 4) may be more appropriate.

We used the half gland for analysis because of a compromise. Overall, in a study with a large number of patients, the most clinically relevant level of analysis is the whole gland: equivalent to the question “have we missed a significant tumor in this *patient.*” However, we had a smaller number of patients scored negative on MRI (*n* = 9/54, 17%) than one would expect in the group of patients with persistently elevated PSA level and a previous negative biopsy. The number of *halves* that scored negative was much higher—39 of 108 (36%) (apparent disease on MRI is often unilateral).

It would have been possible to analyze the prostate at the level of a number of smaller quadrants, but this has a number of drawbacks. Firstly, boundary effects increase, so that complicated rules must be devised to account for imperfect registration of MRI and biopsy or prostatectomy specimens. Secondly, the *clinical question* is usually at the level of the prostate, or half gland—“does this patient have disease?” or “do I only need to treat half the gland?” Quadrant analysis results in a spurious apparent increase in specificity [15] and is difficult to interpret clinically.

### 4.1 Previous studies

Two important parameters have been measured in previous studies: the overall detection rate of cancer in men with a previous negative biopsy, and the proportion of tumors lying anteriorly. A targeted biopsy technique, as used in almost all previous papers, precludes the estimation of sensitivity and specificity.

For the question of the prevalence of tumor in this group, our finding of any cancer in 63% and significant in 43% is similar to several previous studies: 40% for any tumor in 1 group of 43 patients [4], 42% for any tumor in men undergoing template biopsy without MRI (*n* = 102) [16], 48% in a recently published study [17], and 59% for significant tumor in another group of patients with suspicious foci on MRI [4,5].

Several groups have provided estimates of the proportion of tumors lying anteriorly: one group using targeted biopsies found that missed tumors were in the “most ventral part of the transition zone” or anterior horns in a total of 68% of patients [5], and another group found (again using targeted cores) that 76% of missed tumors lay in the transition zone [4]. Finally, a group using template biopsies but not MRI found that 59% of tumors lay in the transition zone or anterior parts of the peripheral zone [16]. Again, these results are in agreement with those obtained using our systematic biopsy technique, where the great majority of patients (92%) had some anterior tumor, and 47% had *only* anterior tumor.

Our finding of a relatively low specificity of MRI if 3/5 is considered positive is consistent with the study by Franiel et al. [18]. Fifty-four patients with at least 1 prior negative prostate biopsy underwent mp-MRI including spectroscopy. MRI-guided biopsies were obtained and cancer was found in 21 of 54 (39%) patients. Out of 178 lesions detected on MRI, 53 were positive on biopsy: a positive predictive value of 30%.

Arumainayagam et al. [19] recently evaluated the performance of mp-MRI in the detection of clinically significant prostate cancer. Their cohort comprised 3 different patient categories; those with known low-risk or low-intermediate-risk prostate cancer (*n* = 51), those with previous negative biopsies (*n* = 10), and those with no prior prostate biopsy (*n* = 3). Two thresholds, definitions 1 and 2, were used to describe clinically significant disease. These are comparable to our UCL definitions 1 and 2, respectively. In ruling out prostate cancer, they had a similar negative predictive value that reached up to 95%.

Perhaps the largest limitation of this study is that the decision to proceed to biopsy was based partly on the MRI findings. This means our estimates of disease prevalence are likely overestimates. However, to some extent this is mitigated by the half-gland analysis and the use of systematic biopsies. At the same time, one would expect to find a low NPV of MRI in a population with a high disease prevalence. However, our NPV was quite high; 79% to 100% for various definitions of clinically significant disease.

Second, although template biopsies perform well for the detection of significant disease (both in theoretical studies and in practice [20], with figures of up to 87% for the detection of significant tumor), they are arguably less accurate than radical prostatectomy, and we are ultimately using a core biopsy technique to estimate tumor significance. Although the technique for doing so has been validated [21], it introduces another source of error.

Third, the scans were interpreted by many different radiologists, and a small minority were performed on a 3 T machine (*n* = 5). This introduces variability in the performance of the diagnostic test, but it does represent real-world experience, and each radiologist was a subspecialist reporting at least 100 mp-MRIs per year.

### 4.2 Clinical implications

Although previous studies have shown that mp-MRI can be used to detect prostate cancer in many men with previously negative biopsies, they have been less effective at answering the question “how reliable is a negative MRI?” because of the targeted biopsy technique that is generally employed. Our estimates of sensitivity suggest that MRI can be very useful for *excluding* tumor in this group, and preventing the need for rebiopsy. This is especially true when the definition of significant disease does not include small tumors or those with a small Gleason 4 component. The low positive predictive value of the test in our study (and in others) implies that MRI cannot be used to reliably infer the presence of disease, but this is not a fundamental drawback if a positive MRI is seen as an indication to biopsy—and though not the subject of this paper, as a map for biopsy as well. It is then acting as a triage test: ruling out disease in some, and triggering biopsy in others.

## 5 Conclusions

Mp-MRI showed good performance at both detection (sensitivity 76%–100%) and ruling out (NPV of 79%–100%) clinically significant disease, according to the definition used. Therefore, we think that mp-MRI has the potential to be used as a triage test in the population with persistently elevated or rising PSA level to select patients that can avoid unnecessary prostate biopsy.

## Figures and Tables

**Table 1:** Detailed MRI scan parameters

	TR	TE	Flip angle, deg	Plane	Slice thickness, mm	Matrix size	Field of view, mm	Time for scan
1. T2 TSE	5,170	92	180	Axial and coronal	3 (10% gap)	256×256	180×180	3 min 54 s (ax), 4 min 18 s (cor)
2. VIBE fat saturation	5.61	2.52	15	Axial	3 (20% gap)	192×192	260×260	7 min (17 s per acquisition)
3. Diffusion (b values: 0, 150, 500, and 1,000)	2,200	Min (<98)		Axial	5	172×172	260×260	5 min 44 s (16 averages)
4. Diffusion (b = 1,400)	2,200	Min (<98)		Axial	5	172×172	320×320	3 min 39 s (32 averages)

**Table 2:** Definitions of clinical significance used as target conditions in the reference test, template prostate mapping

Definition of clinically significant prostate cancer	Maximum cancer core length (CCLmax)	Gleason score
UCL definition 1	≥6 mm or ≥4 + 3, or both	
UCL definition 2	≥4 mm or ≥3 + 4, or both	
Gleason score ≥4 + 3	Any	≥4 + 3
Gleason score ≥3 + 4	Any	≥3 + 4
CCLmax ≥6 mm	≥6 mm	Any
CCLmax ≥4 mm	≥4 mm	Any

**Table 3:** Baseline demographics of 54 men, with prior negative TRUS biopsy, undergoing mp-MRI followed by template prostate mapping

Age, y (median, range)	64 (39–75)
PSA (median, range)	10 (2–23)
Prostate volume (median, range)	53 (19–136)
Number with no cancer on TPM, *n* (%)	20/54 (37%)
Number with cancer on TPM, *n* (%)	34/54 (63%)
Number of biopsies at TPM (median, range)	45 (21–137)

Gleason score on TPM, *n* (%)	
6	16/34 (47%)
7 (3 + 4)	13/34 (38%)
7 (4 + 3)	5/34 (15%)
≥8	0/34 (0%)

**Table 4:** Correlation between different MRI scores and systematic template mapping biopsy findings based on Gleason grade

	No cancer	Gleason 3 + 3	Gleason 3 + 4	Gleason 4 + 3	Total
MRI score 1	0	0	0	0	0
MRI score 2	26	10	3	0	39
MRI score 3	23	9	2	1	35
MRI score 4	7	7	5	4	23
MRI score 5	1	2	8	0	11
Total	57	28	18	5	108

**Table 5:** The performance characteristics of mp-MRI with a radiological score of ≥3 to detect and rule out clinically significant cancer on TPM at multiple levels of significance (95% confidence intervals in parentheses)

Classification	ROI	TP	FN	TN	FP	SENS	SPEC	PPV	NPV
UCL2	108	26	8	31	43	76	42	38	79
						(59−91)	(30−52)	(25−49)	(66−92)

**Table 6:** The performance characteristics of mp-MRI with a radiological score of ≥3 to detect and rule out clinically significant cancer on TPM at multiple levels of significance (95% confidence intervals in parentheses)

Classification	ROI	TP	FN	TN	FP	SEN	SPEC	PPV	NPV
UCL1	108	18	2	37	51	90	42	26	95
						(74−100)	(30−50)	(16−36)	(86−100)
Gleason 4 + 3	108	5	0	39	64	100	38	7	100
						(100−100)	(28−47)	(2–14)	(100−100)
Gleason 3 + 4	108	20	3	36	49	87	42	29	92
						(70−100)	(31−52)	(19−40)	(82−100)
CCLmax ≥6	108	16	2	37	53	89	41	23	95
						(71−100)	(30−51)	(14−33)	(86−100)
CCLmax ≥4	108	20	7	32	49	74	39	29	82
						(54−90)	(28−49)	(19−39)	(69−93)
Any cancer	108	38	13	26	31	74	45	55	66
						(61−86)	(31−59)	(43−67)	(51−82)

**Table 7:** The performance characteristics of mp-MRI with a radiological score of ≥4 to detect and rule out clinically significant cancer on TPM at multiple levels of significance (95% confidence intervals in parentheses)

Classification	ROI	TP	FN	TN	FP	SENS	SPEC	PPV	NPV
UCL2	108	23	11	63	11	67	85	67	85
						(50−83)	(76−93)	(48−84)	(77−93)
UCL1	108	16	4	70	18	80	80	47	94
						(60−91)	(71−88)	(31−64)	(89−99)
Gleason 4 + 3	108	4	1	73	30	79	71	12	99
						(29−100)	(62−79)	(2−23)	(95−100)
Gleason 3 + 4	108	17	6	68	17	74	80	50	92
						(55−90)	(71−89)	(32−69)	(85−97)
CCLmax ≥6	108	14	4	70	20	77	78	41	94
						(57−95)	(69−86)	(26−59)	(89−99)
CCLmax ≥4	108	17	10	64	17	62	79	49	86
						(41−80)	(70−88)	(32−68)	(78−93)
Any cancer	108	26	25	49	8	51	86	76	66
						(38−65)	(76−95)	(61−91)	(55−77)
